# Supplementary material for: Whole Exome Sequencing in Multi-Incident Families Identifies Novel Candidate Genes for Multiple Sclerosis
Source: Int J Mol Sci. 2022 Sep 28;23(19):11461. doi: 10.3390/ijms231911461 (PMC9570223; doi:10.3390/ijms231911461)
Supplement: Supplementary file 1 [file ijms-23-11461-s001.zip › Horjus et al. Figure S1 Table S1 and Table S3.pdf]

Figure S1. Pedigrees with 18 rare variants of the nine multi-incident MS families. For each variant, the genotype is presented for each family member.

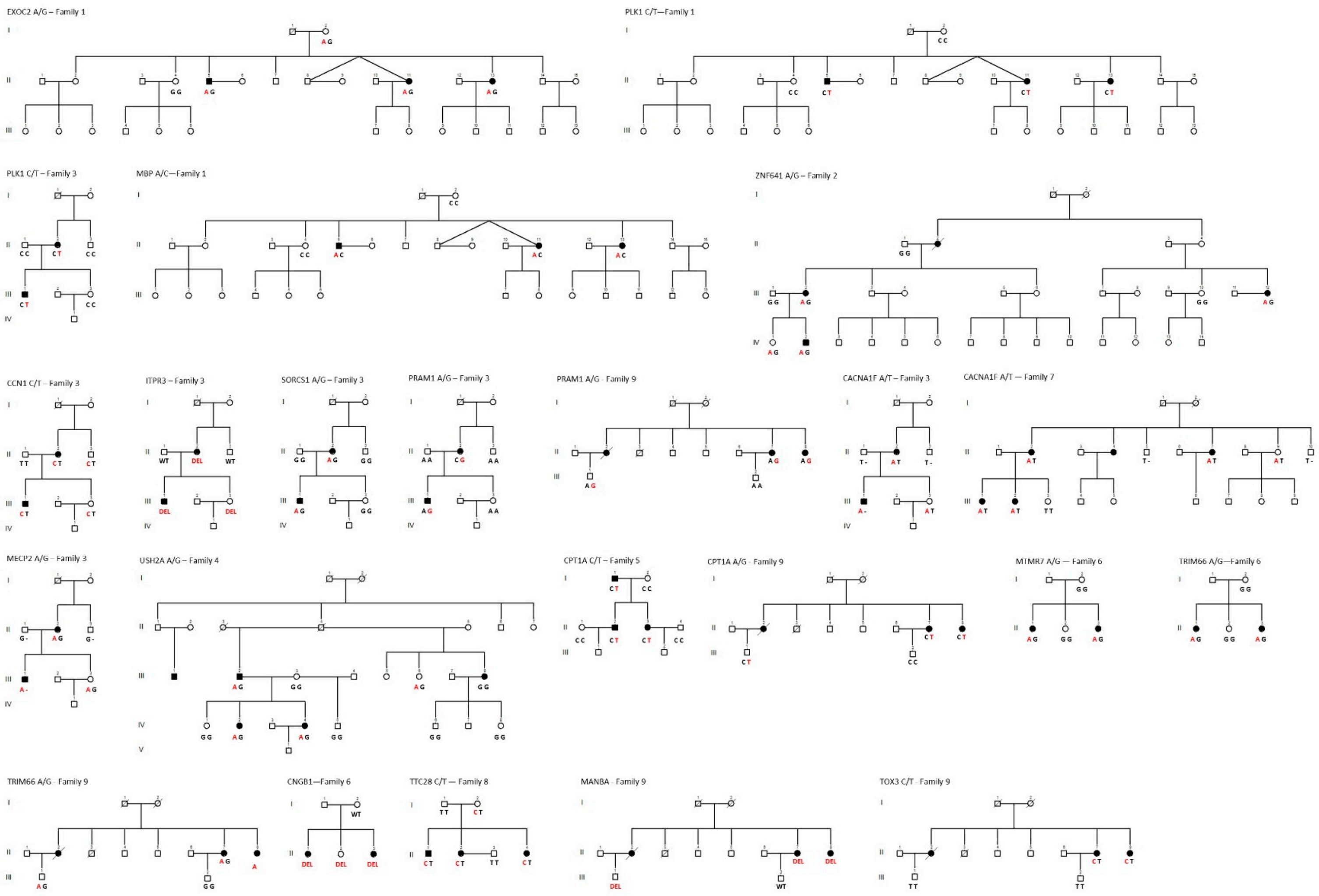

Table S1. An overview of the selection criteria that each of the 18 rare genetic variants meets is provided. If a variant or gene had a significant P-value in either the exome chip study [24] or based on the results from the genome-wide association study (GWAS) [23] – i.e. the gene-wide P-value – by the International Multiple Sclerosis Genetics Consortium (IMSGC), said P-value is provided.

Gene	Variant ID	Amino Acid Change	Present in 2/9 Families	Literature Link(s) with MS	*p*-Value Exome Chip	Gene-Wide *p*-Value
EXOC2	rs760365995	p.Pro134Leu				2.50×10^{-3}
PLK1	rs35056440	p.Leu261Phe	✓			
MBP	18:74696828	p.Gly151Val		✓ [37,38]		
ZNF641	rs200502528	p.His342Tyr				3.76×10^{-2}
CCNI	rs139547927	p.Tyr334Cys				1.46×10^{-3}
ITPR3	6:33648383–33648394	-(DEL (a))				2.65×10^{-5}
SORCS1	10:108439392	p.Ser554Leu				3.74×10^{-2}
PRAM1	rs138042924	p.Ser387Pro	✓		3.26×10^{-2}	3.55×10^{-4}
CACNA1F	X:49067910	p.Met1324Leu	✓			
MECP2	rs61751445	p.Thr311Met		✓ [39,40]		
USH2A	rs200802261	p.Pro2870Leu			2.76×10^{-2}	
CPT1A	rs140958507	p.Arg288Gln	✓	✓ [41]		
MTMR7	rs760803217	p.Thr6Met				2.83×10^{-2}
TRIM66	rs138444298	p.Arg1037Trp	✓			
CNGB1	rs776392588	-(DEL (b))				
TTC28	22:28501278	p.Tyr1099Cys				3.15×10^{-2}
MANBA	rs1283991082	-(DEL (c))				
TOX3	16:52580565	p.Tyr24Cys				3.07×10^{-2}

(a) Deletion of 12 nucleotides: GTGCTGAGCGTT>-

(b) Deletion of 7 nucleotides: AAGTAGT>-

(c) Deletion of 15 nucleotides: GAGCAAAGAAATTCT>-

Table S3. An overview of the sequences of the forward and reverse primers that were used for the Sanger sequencing and their optimal annealing temperatures in order to validate the 18 variants in the family members of the multi-incident MS families.

Family	Chr	Gene	Variant ID	Forward Primer	Reverse Primer	Optimal Annealing Temperature
1	chr6	*EXOC2*	rs760365995	5′ TTCCTGGGATGTTTCTGGAG	5′ CCAACAGGCATTTTGTATTAGG	59 °C
1	chr16	*PLK1*	rs35056440	5′ AGGATGCCTGACCTTTGTTC	5′ GGTGGGATAGACCTATTTCTGG	59 °C
1	chr18	*MBP*	18:74696828	5′ GAAATCCACGCGTAAATGC	5′ TGAACCACTCACCAGTCCAG	59 °C
2	chr12	*ZNF641*	rs200502528	5′ TCAGCCAGTGTCTCACAAGG	5′ GGCGAAGACATCACCTCATC	59 °C
3	chr4	*CCNI*	rs139547927	5′ AGAAACAGGCTGCAAAGGTG	5′ GCCCAGACTTCTCCAAGGAC	59 °C
3	chr6	*ITPR3*	6:33648383–33648394	5′ AGGAGGTGTGGGAGTGGAG<br>5′ AGGAGGTGTGGGAGTGGAG	5′ ACCTGCAGGGAAGTGCTG<br>5′ CTAGGCAAGTCGGCCCTG	56 °C<br>58 °C
3	chr10	*SORCS1*	10:108439392	5′ GGTTTTCTGCAGACTTGCTTC	5′ TATTTGGGGGATGGAAGATG	59 °C
3	chr16	*PLK1*	rs35056440	5′ AGGATGCCTGACCTTTGTTC	5′ GGTGGGATAGACCTATTTCTGG	59 °C
3	chr19	*PRAM1*	rs138042924	5′ CACCCACCTATGGATGCTG	5′ AGTCAGCGTGCTTCCCAAG	59 °C
3	chrX	*CACNA1F*	X:49067910	5′ GTGGTGGTTGTGAGGAAATG	5′ ATCTCCTCATTCCCCAGTCC	59 °C
3	chrX	*MECP2*	rs61751445	5′ TCTGAGTGGTGGTGATGGTG	5′ CAGGTCATGGTGATCAAACG	59 °C
4	chr1	*USH2A*	rs200802261	5′ CGCTCACACAATGAAGACAC	5′ GTGCGAAACTGGCAGTAAAG	59 °C
5	chr11	*CPT1A*	rs140958507	5′ CCCGGCCACAAACTGTATATT	5′ CAAAGTCAAAGTGGAGAAAATCA	59 °C
6	chr8	*MTMR7*	rs760803217	5′ CAGCAGCAGCTAAGGCTAGG	5′ CAGCCTGTACTCCCCATCTC	59 °C
6	chr11	*TRIM66*	rs138444298	5′ CAAGCCAGAGGAAAACTGC	5′ CCAAGCCTGTGGCTAGAAAG	59 °C
6	chr16	*CNGB1*	rs776392588	5′ TCCGATCATCACAGAGAAAGC	5′ TCTGTCTTTTGCACAACATGC	58 °C

7	chrX	*CACNA1F*	X:49067910	5′ GTGGTGGTTGTGAGGAAATG	5′ ATCTCCTCATTCCCCAGTCC	59 °C
8	chr22	*TTC28*	22:28501278	5′ CTGAGACCCTTACTCCACACAG	5′ CCTTGAACGCCAGCTGAAC	59 °C
9	chr4	*MANBA*	rs1283991082	5′ CTTCGATCTTAGAAAACAATCC	5′ GCCTCTCTGACTACTTGACC	56 °C
9	chr11	*TRIM66*	rs138444298	5′ CAAGCCAGAGGAAAACTGC	5′ CCAAGCCTGTGGCTAGAAAG	59 °C
9	chr11	*CPT1A*	rs140958507	5′ CCCGGCCACAAACTGTATATT	5′ CAAAGTCAAAGTGGAGAAAATCA	59 °C
9	chr16	*TOX3*	16:52580565	5′ GCCAAAGAAAAGTGCAGCAG	5′ CTCGACGGTGGATCTAGCG	59 °C
9	chr19	*PRAM1*	rs138042924	5′ CACCCACCTATGGATGCTG	5′ AGTCAGCGTGCTTCCCAAG	59 °C
